# Purification and Characterization of a Nonspecific Lipid Transfer Protein 1 (nsLTP1) from Ajwain (*Trachyspermum ammi)* Seeds

**DOI:** 10.1038/s41598-019-40574-x

**Published:** 2019-03-11

**Authors:** Meshal Nazeer, Humera Waheed, Maria Saeed, Saman Yousuf Ali, M. Iqbal Choudhary, Zaheer Ul-Haq, Aftab Ahmed

**Affiliations:** 1 Department of Biomedical and Pharmaceutical Sciences, Chapman University School of Pharmacy, Irvine, CA 92618 USA; 2 H.E.J. Research Institute of Chemistry, International Center for Chemical and Biological Sciences, University of Karachi, Karachi, 75270 Pakistan; 3 Dr. Panjwani Center for Molecular Medicine and Drug Research, International Center for Chemical and Biological Sciences, University of Karachi, Karachi, 75270 Pakistan

## Abstract

Ajwain *(Trachyspermum ammi)* belongs to the family Umbelliferae, is commonly used in traditional, and folk medicine due to its carminative, stimulant, antiseptic, diuretic, antihypertensive, and hepatoprotective activities. Non-specific lipid transfer proteins (nsLTPs) reported from various plants are known to be involved in transferring lipids between membranes and in plants defense response. Here, we describe the complete primary structure of a monomeric non-specific lipid transfer protein 1 (nsLTP1), with molecular weight of 9.66 kDa, from ajwain seeds. The nsLTP1 has been purified by combination of chromatographic techniques, and further characterized by mass spectrometry, and Edman degradation. The ajwain nsLTP1 is comprised of 91 amino acids, with eight conserved cysteine residues. The amino acid sequence based predicted three dimensional (3D) structure is composed of four α-helices stabilized by four disulfide bonds, and a long *C*-terminal tail. The predicted model was verified by using different computational tools; i.e. ERRAT, verify 3D web server, and PROCHECK. The docking of ajwain nsLTP1 with ligands; myristic acid (MYR), and oleic acid (OLE) was performed, and molecular dynamics (MD) simulation was used to validate the docking results. The findings suggested that amino acids; Leu11, Leu12, Ala55, Ala56, Val15, Tyr59, and Leu62 are pivotal for the binding of lipid molecules with ajwain nsLTP1.

## Introduction

Lipid transfer proteins (LTPs) are puzzling plant proteins because they are not only involved in lipid transfer but in many other functions, which make them unique. These proteins are present throughout the plant kingdom in abundance. In plants, non-specific lipid transfer proteins (nsLTPs) are known to be involved in nonspecific exchange of various lipids and hydrophobic molecules between the membranes. These lipid transferring plant proteins reversibly bind and transfer a range of lipids, such as fatty acids (having 10–19 carbons), phosphatidylglycerol, galactolipids, phospholipids, and fatty acyl-coenzyme A^1–5^. In plants, LTPs are functionally important in resistance to stress, sexual reproduction, seed development, germination, plant defense against pathogenic attack, cuticle formation, and pollen tube adhesion^6–11^. Their role in the on-set of food and pollen allergy in human is also reported due to their high resistance against digestive enzymes, and heat treatment^12,13^. Large numbers of LTPs from both mono-cotyledon, and di-cotyledon plant species (fruits, vegetables, nuts, and cereals) have been reported including; barley aleurone, spinach leaves, wheat, barley, maize leaves, corn seeds, cumin seeds, mung beans, onion seeds, castor beans, carrot embryos, tomato stems, rice, Chinese cabbage, arabidopsis, barrel medic, eggplant, peach, apricot, orange, apple, cherry, lemon, grape, tomato, strawberry, lettuce, cabbage, walnut, hazelnut, and olives^14–21^. The nsLTPs found in plants comprise 4% of their total soluble proteins. They are highly soluble and exhibit high isoelectric points (*pI*) that ranges between 8.0–10.0 due to the presence of many charged amino acids^22^. The plant LTPs can be purified from soluble crude proteins by combination of different chromatographic techniques, such as gel filtration chromatography (GFC), cation exchange chromatography, and reverse-phase high performance liquid chromatography (HPLC)^23,24^.

The LTPs have conserved eight cysteine residues, forming four disulfide bridges, known to be as eight-Cys motif (8CM). The general form of 8CM is CXn-C-Xn-CC-Xn-CXC-Xn-C-Xn-C, which is stabilized by very compact four or five, α-helices^2,4^. Due to very compact structure, LTPs are resistant to heat, salt, proteases, and other denaturing agents^25–27^. LTPs can be classified into ten classes; five of them known as major types, and five minor types. Major types includes: LTP1, LTP2, LTPc, LTPd, and LTPg, while minor types include: LTPe, LTPf, LTPh, LTPj, and LTPk. The classification of LTPs depends on sequence identity, the spacing between 8CM, and also upon conserved introns^4,5,28,29^.

In flowering plants, the most abundant LTPs are LTP1, and LTP2. These LTPs are not found in mosses, non-seed plants, and liverworts. These findings suggested that these were most probably the first LTP types evolved in land plants. These two structurally related families of proteins have low sequence homology, but exhibit similar folding properties^4,5,28,30^. LTPs1 have molecular weight of approximately 9–10 kDa and possess a long tunnel-like cavity for substrate binding, while LTPs2 have a molecular weight of approximately 7 kDa, and possess two adjacent hydrophobic cavities for substrate binding^31,32^. The disulfide bonding signature between cysteine residues is different in both classes. In LTP1, disulfide bonding signature is Cys_1_-Cys_6_, Cys_2_-Cys_3_, Cys_4_-Cys_7_, and Cys_5_-Cys_8_, while in LTP2 it is Cys_1_-Cys_5_, Cys_2_-Cys_3_, Cys_4_-Cys_7,_ and Cys_6_-Cys_8_^4,5,25,33–35^. The precursor nsLTPs has an *N*-terminal signal peptide of 20–25 amino acid residues. Upon removal of this signal peptide, mature protein is either secreted outside the cell as apoplastic protein or bound to the cell wall or plasma membrane^14,23,36^. Recently, it is also reported that apoplastic nsLTP from germinating sunflower seeds undergo endocytosis and relocalization into intracellular organelles involved in lipid metabolism^37^. nsLTP genes are found to be upregulated in response to many pathogenic fungal infections in barley, radish, onion, sugar beet, and sugar beet leaves^38^. Similarly, in tobacco and *Arabidopsis* leaves, up-regulation of barley LTP2 transgene was observed against bacterial pathogens^31^. Many plant LTPs, such as mung bean, wheat, coffea, *Arabidopsis*, spinach, and pepper known to be active against bacterial, and fungi strains^39–42^. LTPs from the seeds of *Brassica campestris* and Chinese daffodil (*Narcissus tazetta*) have shown the ability to inhibit human tumor cells proliferation^43,44^. Due to their high stability and wide binding properties, they can also protect drugs from oxidation and degradation and therefore, can also be used as effective drug delivery systems^27,28^. The plant LTPs ability to bind different lipids make them feasible candidates for drug delivery systems^44^. The wheat LTP1 is known to bind with many drugs used to treat HIV-1, leishmania, and fungi species^20,21,44–47^.

In recent years various papers have been published to explore the structure and functions of LTPs^4,5,14^. This has been observed by X-rays, nuclear magnetic resonance (NMR), 3D structure, and MD simulation^4,5,14^. These techniques have been used to explore the structure of free state protein, as well as complex with ligands (lipid molecules)^20^. All techniques suggested that LTPs consisted of 4 α-helices, long *C*-terminal tail, and four sulphide bridges forming a compact three dimensional structure^23,28^.

In traditional and folk medicine (Sino Tibetian, Unani, and Ayurveda systems), spices and herbs have been used to treat various diseases. *Trachyspermum ammi*, generally known as ajwain or carom, belongs to the Umbelliferae family (also called Apiaceae). It is commonly grown in Pakistan, India, Afghanistan, Iran, and in the European region. The grayish brown seeds are grown under semi-arid and arid regions that are rich in salts^48^. The ajwain seeds are used in traditional medicine due to their carminative, diuretic, antiseptic, laxative, hepatoprotective, antiviral, antibacterial, antioxidant, and antihypertensive properties^49,50^. It is also used for flatulence, atonic dyspepsia, diarrhea, and is often recommended for cholera^51,52^.

Computational methods to predict 3D protein structure and ligand-protein binding interaction have played an important role in protein structural-functional studies in biochemical research and proteomics over the past decade. In our present work, we reported the characterization of nsLTP1 from *Trachyspermum ammi* (ajwain) seeds for the first time. We built a 3D structure for nsLTP1 using homology modeling approach. Computational tools like homology modelling and molecular docking simulation were utilized to find out the best possible 3D structure of ajwain nsLTP1 and its mode of interaction with fatty acid molecules. Moreover, ajwain nsLTP1 conformational changes induced by ligands during the MD simulations were observed. Root mean square deviation (RMSD), root mean square fluctuation (RMSF), and secondary structure prediction (DSSP) analysis tool were applied to check the stability of the apo and complex systems during 10 ns of the simulated time. It was revealed that fatty acid molecules can accommodate with its hydrophobic properties in the hydrophobic tunnel of ajwain nsLTP. As there is an increasing interest in nsLTPs due to their vital role in plant physiology, this study will be expected to enhance further knowledge about their structure-function relationship.

In summary, our results demonstrate a new addition of member in plant LTPs family, ligand binding interactions with ajwain nsLTP1, can develop a better understanding about its biological functions. It might shed light on future pharmacological or medical industrial applications.

## Results

### Purification of nsLTP1 protein from ajwain seeds

**Figure 1:**
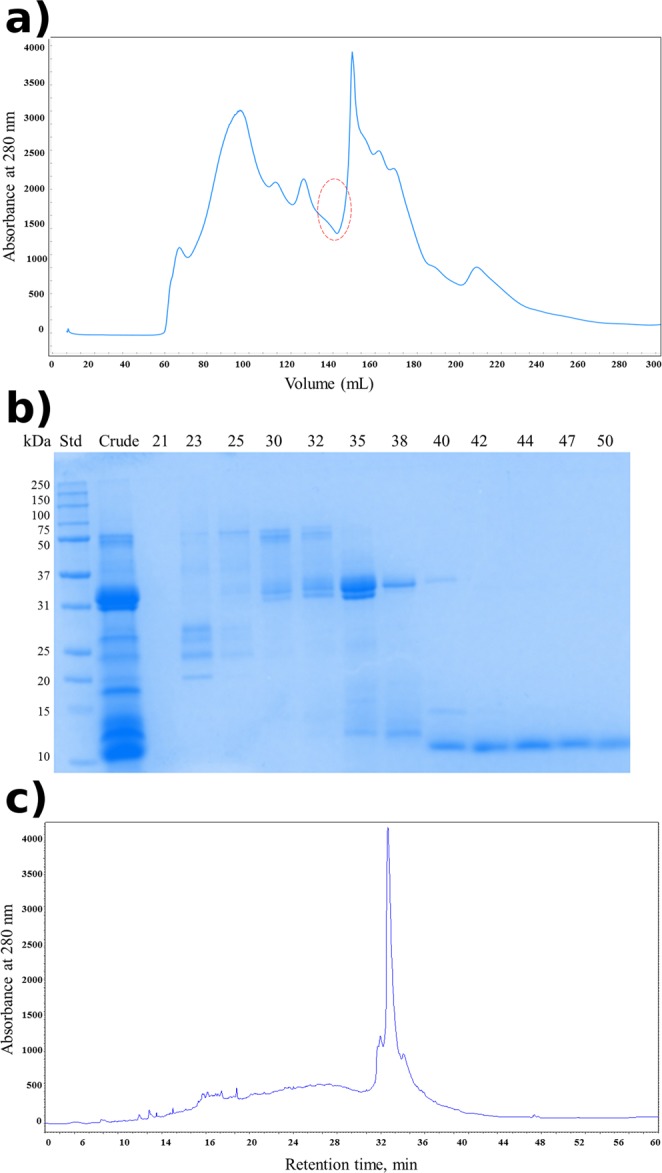
Purification of ajwain nsLTP1. (**a**) Fractionation profile of ammonium sulfate precipitated proteins from ajwain (*Trachyspermum ammi)* on Sephacryl S-200 (2.6 × 60 cm) column. Red circle represents the fractions containing nsLTP1. (**b**) Electrophoretic profile by Tris/Tricine SDS-PAGE (12%) of ajwain seeds proteins precipitates and gel filtration chromatography fractions. Lane 1, standard molecular weight marker Std, Lane 2, crude proteins, C, and Lane 3–14, GFC fractions 21, 23, 25, 30, 32, 35, 38, 40, 42, 44, 47, and 50 (**c**) Separation profile of gel filtration pooled fractions (42–50) containing ns-LTP1 protein by RP-HPLC.

The proteins from defatted ajwain seeds in the n-hexane were extracted in 20 mM Tris/HCl buffer pH 8.0. The proteins were successfully recovered from the extract using ammonium sulfate precipitation. The gel filtration column equilibrated with 20 mM Tris/HCl buffer, pH 8.0 was employed as a first dimensional chromatography. Elution profile (Fig. 1a) clearly showed that proteins are separated based on their molecular mass. The SDS-PAGE analysis of separated gel filtration fractions and crude precipitates were achieved by using 12% Tris/Tricine gel. The electrophoretic profile (Fig. 1b) of the gel filtration fractions (42–50) clearly showed a single band at ~10 kDa. These fractions were pooled, concentrated and further purified by RP-HPLC using an Aeries Widepore C4 (250 × 4.6 mm) column. The chromatogram observed revealed a major peak eluted at a retention time of 32 min (Fig. 1c).

### Complete primary sequence of purified ajwain nsLTP1

**Figure 2:**
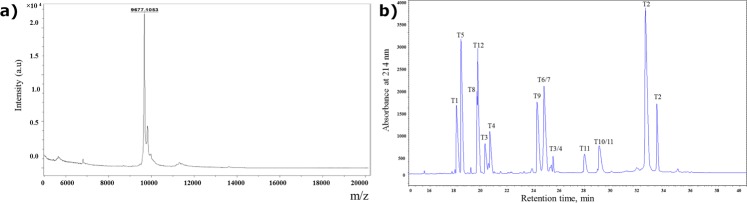
(**a**) MALDI-TOF mass spectra of purified aiwain nsLTP1 protein (**b**) RP- HPLC separation profile of tryptic digest of ajwain nsLTP1. Column Aeris Peptide C18 (4.6 × 2 50 mm), gradient 0–60% B in 40 min. Absorbance was monitored at 214 nm.

The complete primary structure of ajwain nsLTP1 has been deduced by using *N*-terminal amino acid sequencing of the intact protein, which allowed the identification of first 32 amino acid residues. The internal protein sequencing was achieved by using the tryptic peptides of alkylated protein by 4-Vinylpyridine. The alkylation of cysteine residues was preferred as nsLTP is rich in cysteine residues and pyridylethylated cysteine (PE-Cys) is easily identified by Edman sequencing technique^53^. Purified protein after alkylation was separated by RP-HPLC using an Aeries Widepore C4 (250 × 4.6 mm) column and protein was eluted at 46 min. MALDI-TOF mass spectrometry analysis of purified nsLTP1 revealed observed mass at 9677.1051 (Fig. 2a). Alkylated protein was digested by enzyme trypsin and peptide fingerprinting performed by using RP-HPLC on Aeries Peptide C18 (250 × 4.6 mm) column (Fig. 2b). The amino acid sequence of intact protein and tryptic peptides showed that the ajwain nsLTP1 consists of 91 amino acids including eight cysteine residues.

### 3D modelling of ajwain nsLTP1

**Figure 3:**
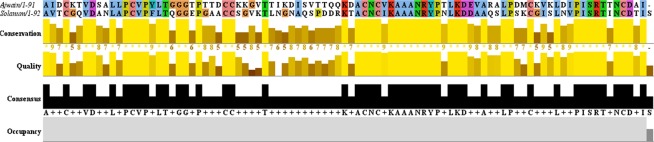
Pairwise protein sequence alignment of Ajwain and Solanum nsLTPs1. The alignment was performed by using Clustal Omega.

**Figure 4:**
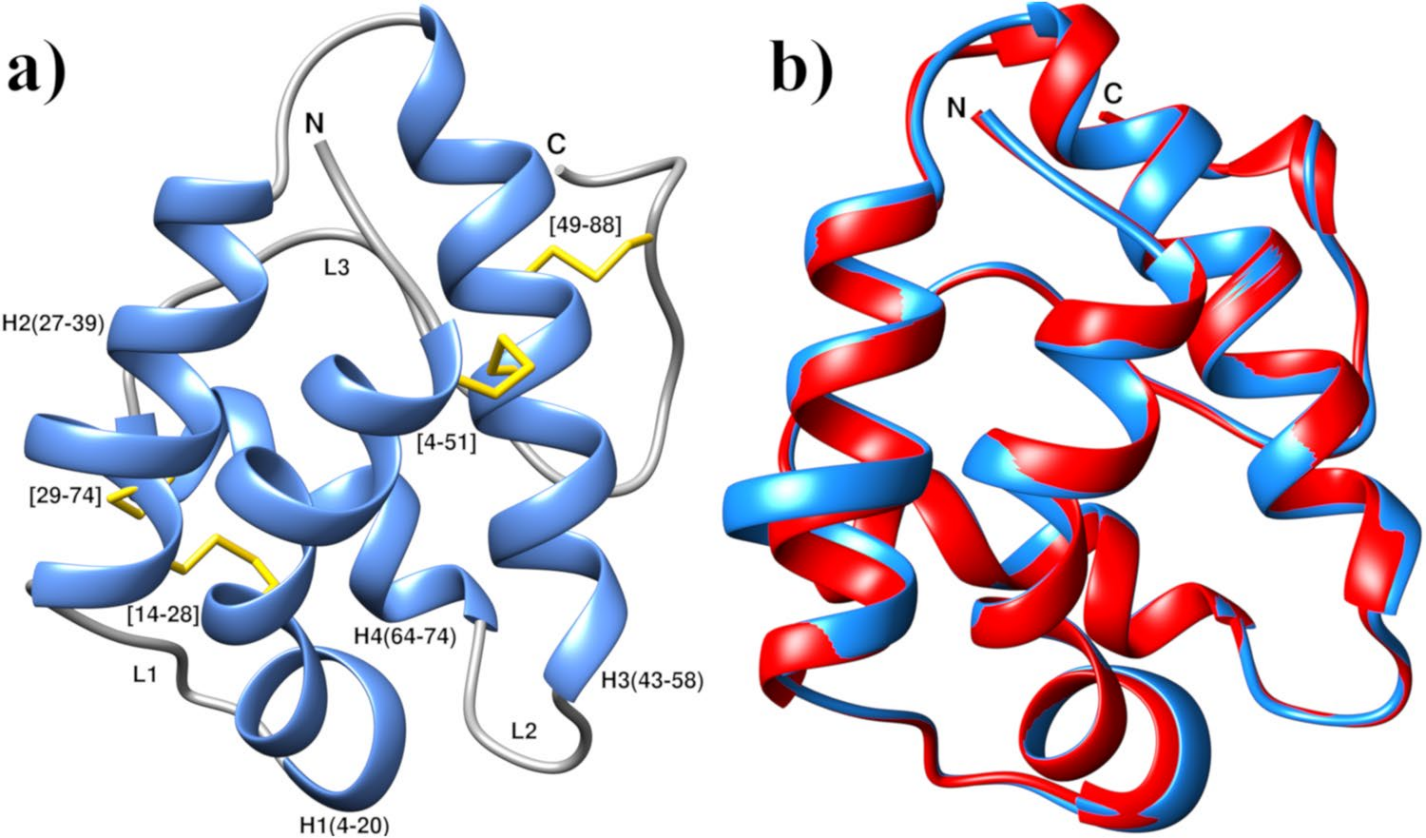
(**a**) Ribbon representation of modeled ajwain nsLTP1. H-represents helix, L-represents loop regions, disulfide bonds are shown as yellow rods. (**b**) Superimposition of refined ajwain nsLTP1 model with template eggplant nsLTP1 (PDB I.D: 5TVI). Blue color represents refined ajwain nsLTP1 model, and red color represent selected homologue 5TVI.

*In silico* modeling is an extremely helpful method for the prediction of 3D structure of protein in drug design. For generation of 3D model of ajwain nsLTP1, a Protein-Protein blast algorithm basic local alignment search tool (BLASTp)^54^ search was performed against protein data bank (PDB)^55^. The *S. melongena* (eggplant)^15^, X-ray crystal structure (PDB ID: 5TVI) was found to be the closest homologue on the basis of optimized E-value with highest sequence identity 52% and similarity 65% determined by the BLASTp Program which was further subjected for template-target alignment by Clustal Omega. The multiple sequence alignment results have been illustrated in Fig. 3. Modeller9v19^56^ was used to generate the 3D structure of ajwain nsLTP1. Best modeled structure was selected on the basis of the minimum discrete optimized protein energy (DOPE) score −8381.56934. The ajwain nsLTP1 consists of four α-helices; H1 (residues 4–20), H2 (residues 27–39), H3 (residues 43–58), H4 (residues 64–74), and a long *C*-terminal loop (residues 74–91). These four α-helices are interlinked by three loops; L1, L2 and L3. The nsLTP1 is stabilized by four disulphide bridges which were observed among Cys4-Cys51, Cys14-Cys28, Cys29-Cys74 and Cys49-Cys88 respectively as illustrated in Fig. 4a. The superposition between atoms of the nsLTP1 homology model over *S. melongena* nsLTP1, yielded a RMSD of only 0.66 Å as depicted in Fig. 4b.

### Molecular docking studies of ajwain nsLTP1

**Figure 5:**
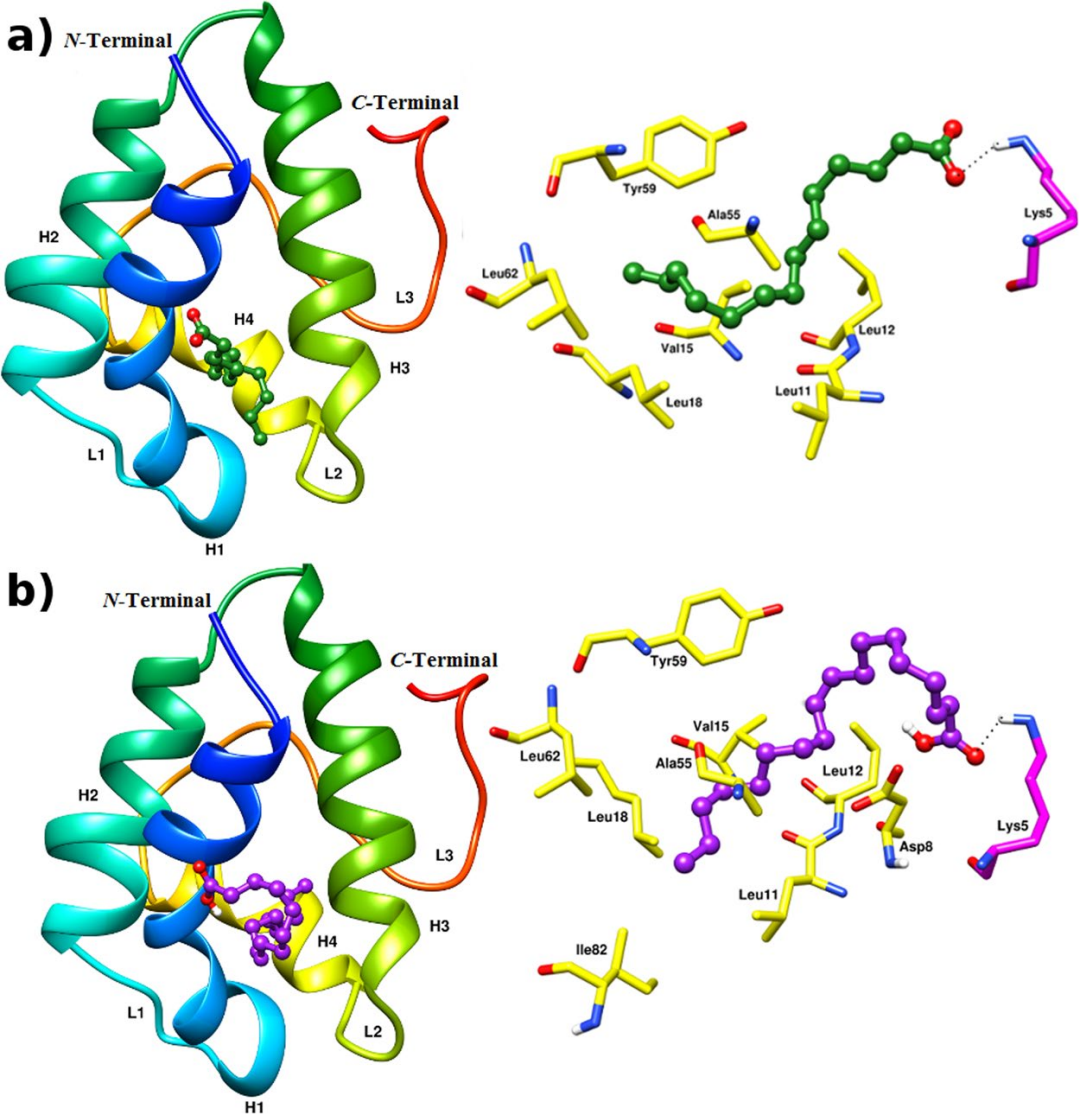
Molecular Docking of lipid molecules with ajwain nsLTP1: (**a**) MYR docked into the active site of ajwain nsLTP1: Residues involved in hydrogen bond (pink) and hydrophobic (yellow) interactions with MYR (green). (**b**) Docking of OLE (purple) inside the cavity of nsLTP1 of ajwain. Residues involved in hydrogen bond (pink), and hydrophobic interactions (yellow) represented for both ligands.

For docking studies, MOE site finder tool was applied to detect functionally potential binding pockets and sub pockets on the surface of the ajwain nsLTP1 protein. It clearly indicates that the residues involved in small molecule binding lies within the helix 1–4 and long C-terminal tail which is the basic feature of all known nsLTPs. The docking analysis of ajwain nsLTP1 complexed with MYR (Fig. 5a) showed lipid molecule was in a tunnel-like hydrophobic pocket surrounded by the *C*-terminal loop and four α-helices. The aliphatic carbons tail of the lipid molecule is buried into the hydrophobic cavity of nsLTP1 while the polar head-group projects out of the binding pocket. MYR showed hydrophobic interaction with, Ala10, Leu11, Leu12, Val15, Leu18, Ala55, Tyr59 and Leu62. A similar lipid binding pattern including Asp8, Ala56 and Ile82 was observed in the ajwain nsLTP1complexed with OLE complex (Fig. 5b). These residues are located in helices H2, H3, at the end of helix H4 and the *C*-terminal loop. Besides that salt bridge was also found with Lys5 in both fatty acid complexes. The ajwain nsLTP1 protein binding with different types of lipid molecules indicates that it can efficiently bind these lipids in its hydrophobic pocket by involving its different residues. From the docking analysis, it was also observed that Asp8, Ala10, Leu11, Leu12, Val15, Leu18, Ala55, Ala56, Tyr59, Leu62 and Ile82 were involved in binding of both the ligands. Therefore, it is observed that these residues are important in nsLTP functioning.

### MD simulations of ajwain nsLTP1

The minimized apo homology modeled ajwain nsLTP1, and its ligand complex systems were subjected to MD simulations. All systems under observation were relaxed through energy minimization by removing steric constraints. The gradual increase of simulation box to body temperature assist in to attain gradual increase in velocity of the system. Newtonian dynamics was used to achieve system equilibration which was succeeded by production run in MD simulations. The dynamical behavior between modeled apo-ajwain nsLTP1 and ajwain nsLTP1-ligand complexes were analyzed during 10 ns of the simulated time. Preliminary analyses of the trajectories such as RMSD, and RMSF, were computed to explore the stabilities of complexes. The simulations were continued till the Cα atoms of the protein were stabilized (equilibrated); however, the complete systems have been equilibrated well within the 70 ps simulation time. RMSD is commonly used to access the dynamic stability of systems as it is a global measure of protein fluctuations.

**Figure 6:**
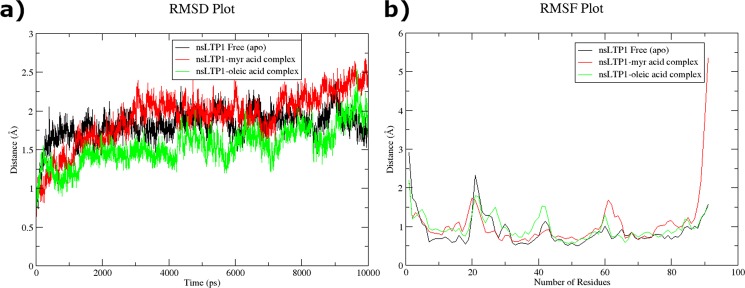
Dynamic behavior analysis of apo, and complexes ajwain nsLTP1. (**a**) RMSD plot analysis of apo and complexes ajwain nsLTP1. (**b**) RMSF plot analysis of apo and complexes ajwain nsLTP1.

The Fig. 6a illustrates Cα backbone atoms RMSD of ligand bound ajwain nsLTP1 complexes in comparison to ajwain nsLTP1 free protein backbone atoms to assess the conformational stability of the systems during the simulations. RMSD of MYR-nsLTP1 complex was initially increased from ∼0.6 to ∼2.1 Å. In the case of OLE-nsLTP1 complex, the RMSD was more stable, showed little change and lied in the average value of about 1.4 Å. When comparing the deviations of nsLTP1 free enzyme with ligand bound complexes, there is no significant fluctuation as apo has the low average RMSD about 1.2 Å. The simulation event showed that the protein structure was perturbed by ligand binding to ajwain nsLTP1 and the oleic-nsLTP1 complex was suggested to be more stable compared to the MYR-nsLTP1 complex system.

**Figure 7:**
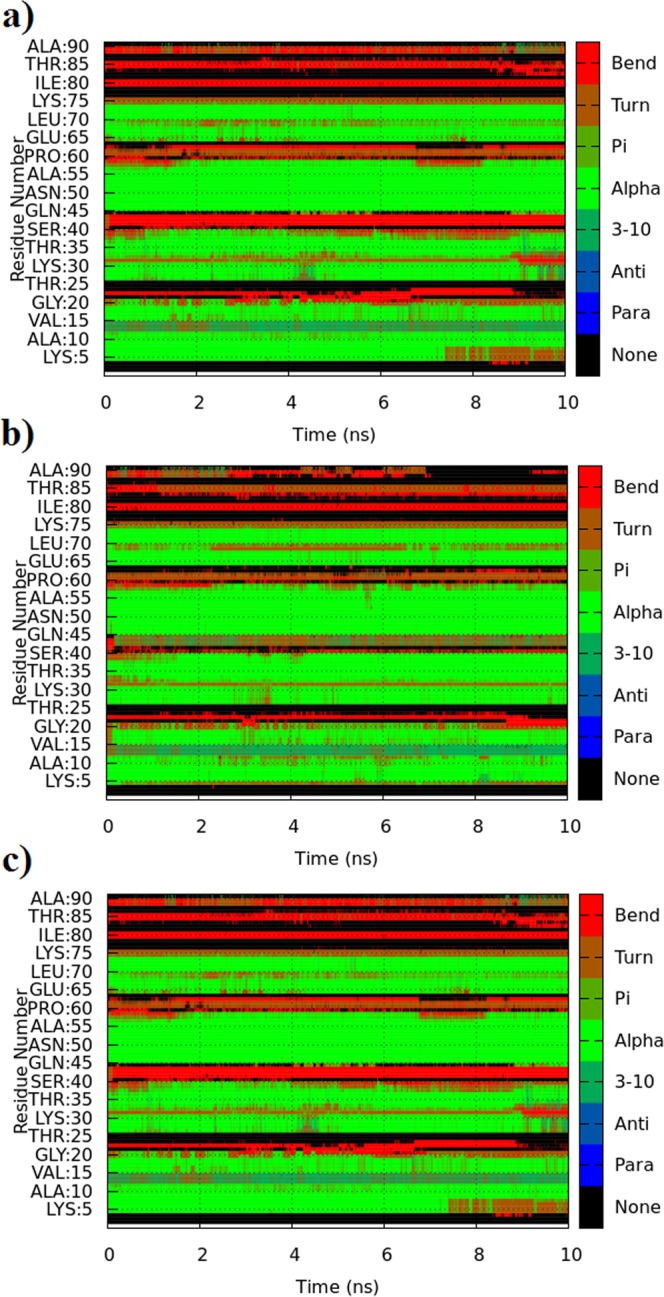
Secondary structure prediction (DSSP) analysis of apo and complex ajwain nsLTP1 protein. (**a**) ajwain nsLTP1 protein apo (**b**) ajwain nsLTP1 complexed with MYR (**c**) ajwain nsLTP1 complexed with OLE.

**Figure 8:**
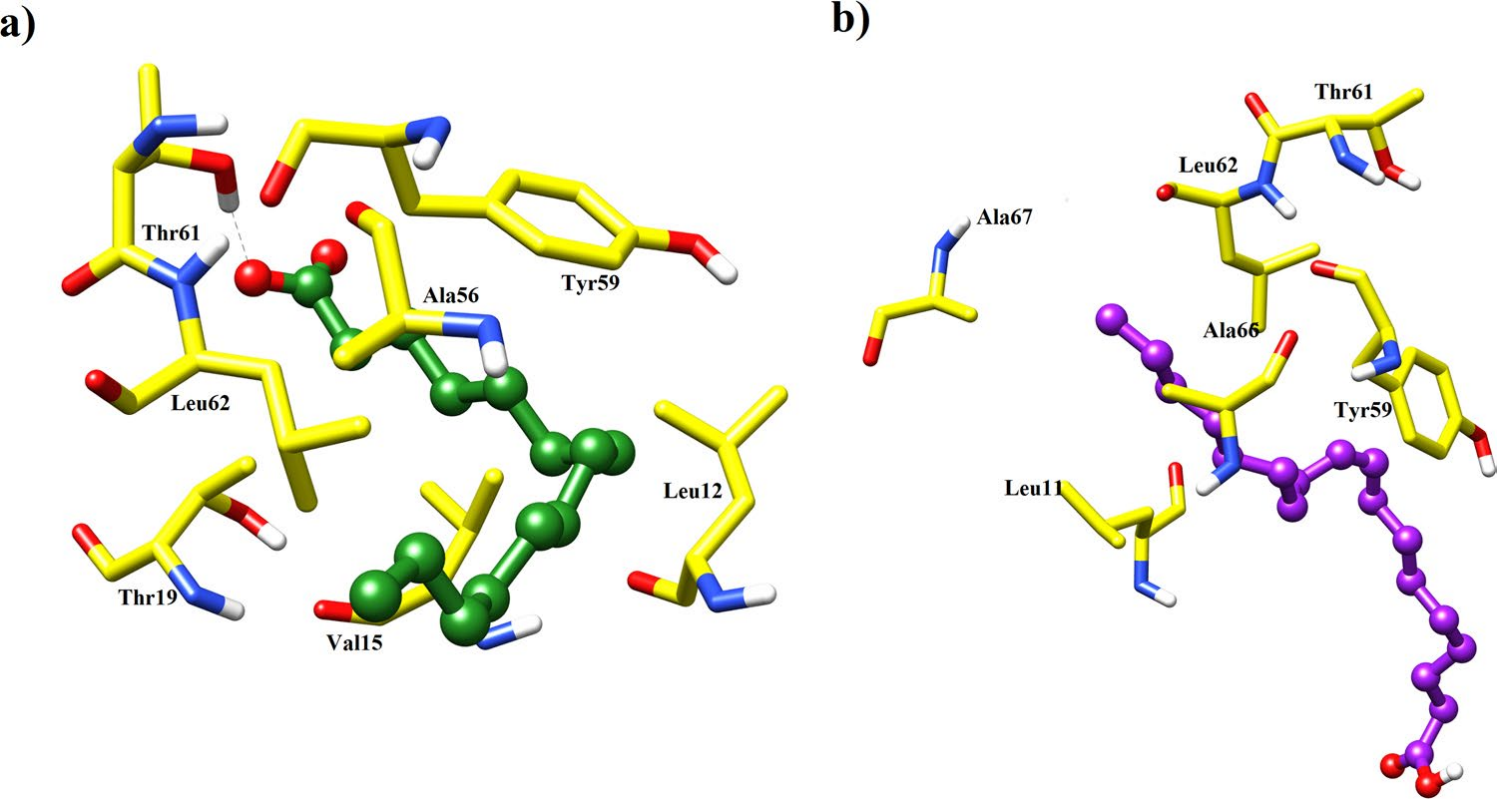
MD simulation of lipid molecules with ajwain nsLTP1 over 10 ns: (**a**) MYR (green) into the active site of ajwain nsLTP1 (**b**) OLE (purple) inside the cavity of ajwain nsLTP1.

This finding was further augmented by computing RMSF of each residue for both complexes in comparison to ligand free system. It clearly demonstrates greater flexibility of residues of the ligand bound systems when compared to the apo enzyme due to ligand binding (Fig. 6b). Secondary Structure analysis of ajwain nsLTP1, and its complexes with MYR and OLE was also performed to observe the perturbed structural differences during simulation with respect to the time of 10 ns (Fig. 7). MD simulation showed that MYR displayed hydrophobic interaction with Leu12, Val15, Thr19, Ala56, Tyr59 and Leu62 and hydrogen bonding with Thr61 at a distance of 1.88 while in case of OLE, amino acids Leu11, Ala56, Thr61, Tyr59, Leu62 and Ala67 showed hydrophobic interaction as depicted in Fig. 8.

## Discussion

The plant nsLTPs are small, cysteine rich, abundant, and highly soluble proteins. The protein was first discovered about 35 years ago. Now this protein family has widely expanded, but still functional expects are undisclosed completely by the biologists. Many functions in plants have been reported i.e. *in vitro* activity against many viruses, fungi, and bacterial strains, ripening of fruits, cuticle layer formation, plant growth^44,57^, etc. The nsLTP family is ubiquitously expressed throughout the plant kingdom. These proteins are known to be expressed in many parts of the plant, such as seeds, fruits, stems, leaves, flowers, roots, etc^14,23,58^. The plant nsLTPs are multigene family of proteins usually encoded by tens of related genes, expressed in different tissues during various developmental stages and physiological stress^5,59^. LTPs are also included in the classification of plant antimicrobial peptides (AMPs). AMPs are small, basic, and cysteine rich peptides produced by plants against pathogens^42,60,61^. In recent years just after their discovery, more than 100 members of the important protein family have been described from about more than 50 different plant species. It is multigenic protein family; in Arabidopsis more than 60 genes, in rice about 77 genes, in maize about 50 genes, and in wheat more than 100 genes have been identified encoding LTPs^21,62,63^.

In the present study we have purified nsLTP1 from very important medicinal plant seeds, ajwain which is also used as a spice because of its aroma throughout the world. The primary structure of ajwain nsLTP1 revealed that it is rich in threonine, alanine, lysine, and aspartic acid residues. The eight cysteine residues form four disulphide bridges which help to stabilize the hydrophobic cavity to transfer lipid molecules. It has highest lysine residues as compared to other known plant nsLTPs. The presence of histidine, tryptophan and phenylalanine residues was not observed. All plant LTPs lack tryptophan^23^ except few isoforms of rice and *Arabidopsis* which have 1–2 tryptophan^42^. Ajwain nsLTP1 also lack tryptophan. The protein consists of several conserved hydrophobic residues including Val7, Leu11, Leu12, Ala55, Ala69, Leu70, Leu78, and Ile82. In the early studies it was reported that the two aromatic residues, Tyr17 and Tyr79 (Tyr81 in maize^64^, and Tyr82 in amaranthus^18^), are known to be as conserved throughout plant LTPs. Later on, in many LTPs, tyrosine (Tyr79) in the *C*-terminus tail was observed to be replaced by phenylalanine, isoleucine, or valine, which suggested that hydrophobic residue in that position is important, not tyrosine. In ajwain nsLTP1; two tyrosine residues Tyr17 and Tyr59 has been observed, but Tyr79 is replaced by aspartic acid. The residue Tyr79 is also absent in *Cuminum cyminum* (cumin)^65^ nsLTP1 and *S. molenga* (eggplant) nsLTP (SM 80.2)^17^. In cumin, Tyr79 is replaced by aspartic acid, and in eggplant it is replaced by asparagine. The aromatic residue at position 79 in plant LTPs is known to be involved in opening and closing of hydrophobic tunnel for the adjustment of the ligand^17^. The X-ray structure of eggplant nsLTP revealed that Asn79 and Val80 are involved in reducing the hydrophobic tunnel, and Val80 performing the same function as Tyr79. In other plant nsLTPs position 80 is occupied by proline^17^. In ajwain nsLTP1 Asp79 and Ile80, are known to be involved in reducing hydrophobic cavities as observed in eggplant nsLTP. Similarly, in the hydrophobic cavity Tyr59 of ajwain nsLTP1 (instead of corresponding residue Ile in rice, hazelnut, wheat, and barley or Val in peach, and maize) is oriented in such a way that it forms hydrophobic interaction with MYR and OLE^17,65,66^. Therefore, in ajwain nsLTP1 Tyr59 is a very crucial residue for ligand binding. The ajwain nsLTP1 has Pro13 in α-helix 1, which is strictly conserved in other plant LTPs. Two other proline residues observed which are also known to be conserved i.e. Pro71, and Pro81^18,64,67^. The protein sequence data reported in this paper will appear in the UniProt Knowledgebase under the accession number C0HLG2.

The BLASTp predicted nsLTP1 (*Solanum molegna*) PDB ID 5TVI as the best template with 52% identity and 65% similarity, 98% query coverage and 2.45E-28 e-value. The Identity score ranges with other reported species of nsLTP between 31–52% sequence identity were observed. According to BLASTp results the highest sequence identity was 52% with *Solanum melogena*, while the least sequence identity for *Hordeum vulgare* nsLTP1 30% was observed. 5TVI was used according to the BLASTp result but to establish confidence on the BLASTp score multiple sequence alignment of the top ranked template sequences (5TVI, 2MAL, 5LQV, 2ALG, 1BWO, 4XUW, 1CZ2, 1FK0, 1UVA, 2N81, 1SIY, 1MID, 1T12) were aligned against the taregeted ajwain sequence illustrated in (Supplementary Table S1 and Fig. S1) while the multiple sequence alignment of best match template 5TVI (*Solanum melogena*) with ajwain (*Trachyspermum ammi)* illustrated in (Fig. 3).

Ajwain nsLTP1 primary structure was used to build 3D model. Refinement of obtained nsLTP1 homology model was validated by ERRAT program^68^. The ERRAT results showed that the overall quality factor for the model is 91.43%. To evaluate the local environment and inter-residue contacts in the model, we used Verify3D web server. Ideally, the 3D-1D profile for each of the 20 amino acids should be in range of 0–0.2. Values less than zero are considered as inaccurate for the homology model. In our case, 85.54% residue having an average score of 0.2 on 3D/−1D profile score^69^, which shows the reliability of the derived model. Also, PROCHECK showed that residues in the predicted model covered 92.4% favorable and 7.6% in additionally allowed regions and no residue observed in disallowed region (Supplementary Fig. S2). It shows the reliability of the predicted model. The stereo-chemical properties of the model were validated by Verify3D web server. The Verify3D plot of ajwain nsLTP1 model showed that the average 3D-1D score of 93.14% residues ≥0.2, positive scores highlight the accurate model folding. The PROSA software^29^ was used to analyze the energy profile of ajwain nsLTP1 model, which showed that there is no positive energy peak, with −4.77 Z-Score. Therefore, it can be inferred that the predicted model is of good quality.

3D structure is a powerful tool used to unlock and disclose biological functions of proteins. The first 3D structure of LTP was revealed in early 1990s and till now, more than 100 LTPs structures are available^59^. All LTPs showed a common feature in that their four helices, and *C*-terminus hydrophobic residues are involved in ligand binding^4,5^. These residues are known to be flexible by attachment of different ligands, to be fit the ligand in the hydrophobic cavity according to the size of ligand. It clearly indicated the plasticity and adaptation of the internal cavity of LTPs upon ligand binding. This capacity is different for every LTP, such as tobacco LTP, which can bind with one type of fatty acid while wheat, eggplant and maize LTPs can bind more than one type of lipid^44^. The binding of one type of lipid is more efficient than the other types of lipids. The fluorescence studies showed, that maize nsLTP1 has more affinity for fatty acids of 16–19 carbon length, which is reduced by the presence of polyunsaturation or hydroxyl groups. Similarly moss, LTPg readily binds to stearoyl-CoA as compared to stearate, and *Arabidopsis* LTP2 binds more efficiently to LPC (lyso- phosphatidylcholine) derivatives that carry longer fatty acyl chains (C18) as compared to shorter acyl chains (C14)^5,70^.

The 3D structure of ajwain nsLTP1 was used for docking by using molecular operating environment (MOE) software package. For this we have selected two ligands, MYR, and OLE as both of them are used in other plant LTPs. The docking indicated that lipid molecules in the hydrophobic cavity surrounded by 4 α-helices and *C*-terminal loop. Aliphatic tail of MYR was projected out from binding pocket. The residues involved with MYR binding are Ala10, Leu11, Leu12, Val15, Leu18, Ala55, Tyr59 and Leu62. Additionally, Asp8, Ala56, and Ile82 are involved in OLE binding as compared to MYR with ajwain nsLTP1 (Fig. 5). In conclusion of docking results, Asp8, Ala10, Leu11, Leu12, Val15, Leu18, Ala55, Ala56, Tyr59, Leu62, and Ile82 were involved in binding of both the ligands. It has been suggested that in plant LTPs, two consensus sequences T/SXXDR/K and PYXIS positioned at about residues 43–47 and 81–85, respectively. In ajwain nsLTP1, first consensus sequence from 43 residues is “TQQKD”, while second consensus sequence from 81 residues is “PISRT”. In the protein Tyr82 is missing, which is known to be involved in ligand binding^4,14,44,65^. As we discussed earlier, that binding pattern involving specific residues with ligands are variables, which has been proved by many solved LTPs 3D structures. In the light of ajwain nsLTP1 docking results Tyr59 is an important residue involved in both ligand binding, and only Ile82 is involved in ligand binding from two consensus sequences. Similarly, two known conserved residues in plant LTPs; involved in ligand binding Tyr17, and Tyr79, in nsLTP1 Tyr17 didn’t show any kind of interaction with any ligand molecules, and Tyr79 is absent.

The MD simulation was performed to analyze dynamic behavior of ajwain nsLTP1 free form as apo, and complexed with MYR, and OLE ligands. The MD simulation is a very powerful technique used to visualize the physical movement of atoms/molecules in fixed time. In one study MD simulation of maize, and barley LTPs was conducted, and observed same results as obtained from experimental data by X-ray, and NMR^71^. This indicated that MD simulation results are very close to experimental data. The MD simulation results concluded that for the most part there are three noteworthy regions of flexibility. The analysis shows that the most flexible part is the loop region; *C*-terminal also has large magnitude of movement and small region of *N*-terminal are also prone to flexibility. The aliphatic carbons tail of the lipid molecule is buried into the hydrophobic cavity of nsLTP1 while the polar head-group projects out of the binding pocket. MYR showed hydrophobic interaction with Ala10, Leu11, Leu12, Val15, Leu18, Ala55, Tyr59, and Leu62. Amino residues 5–8 turn into bend after 7 ns of the simulated time in oleic acid and apo form but in case of MYR complex there was no change observed in this region. Amino acid residues 40–45 turn into helical conformation in case of MYR but in case of apo and oleic acid complex it obtained a bend conformation throughout 10 ns of the simulated time. Amino acid residues 82–85 of a nsLTP1 located in loop region comprised mostly of bends but adopt the β turn conformation after a period of 2 ns due to the complex formation of MYR. There was no structural change observed in this case of OLE binding with the nsLTP1 and nsLTP1 free form. There were minor deviations in the secondary structure of the residues 25–35, 55–60, and 85–90 which showed random transitions from bends/turns to unstructured loops in both complexation and free states. The binding mode of MYR and OLE showed that they easily bind into the hydrophobic cavity of ajwain nsLTP1, as observed in molecular docking and further validation by MD simulation studies. It is proved through structure level analysis that ajwain nsLTP1 can transfer saturated MYR, as well as unsaturated OLE fatty acid.

## Conclusion

In recent years, a number of nsLTPs have been isolated and characterized from various plants. The plants nsLTPs play an important role, not only as a major protein responsible for lipid transport in plants but also for its various physiological and biological functions. The plant nsLTPs are well-known for their antibacterial, and anti-cancer activities. This study revealed that ajwain nsLTP1 binding with various ligands (MYR and OLE), have possible application in drug delivery application. They appeared to be ideally naturally designed moieties which can not only bind and carry lipid molecules but other organic molecules also. These properties made them also suitable for biotechnological applications. This study elaborated the evolutionary relationship of ajwain nsLTP1 with other reported plant LTPs, as well as its potential lipid transfer activity. Subsequently, our *in silico* investigation presents 3D modeling, docking patterns, as well as dynamics interactions of ajwain nsLTP1 with different ligands. This complementary approach of structure, function relationship might be used as foundation for further studies. The coordinate file of ajwain nsLTP1 is submitted to the online protein model database (PMDB)^72^. The PMDB ID of the submitted model is PM0081852.

## Materials and Methods

### Extraction of proteins

The crude proteins extraction from ajwain seeds was achieved by previous methods with slight modifications^23,65^. The seeds of ajwain (*Trachyspermum ammi)* were purchased from the local market in Pakistan. Whole seeds were grinded and defatted by using hexane (1:6, w/v) for 24 hrs. The solid mass of ajwain was recovered by filtration and hexane traces were removed by drying overnight under the fume-hood. The defatted ajwain powder (100 gm) was soaked in 20 mM Tris/HCl, pH 8.0, (1:8, w/v) for 24 hr at 4 °C with continuous stirring. The sample was centrifuged at 14000 rpm for 15 min at 4 °C. The supernatant was collected and protein precipitation was performed by using ammonium sulfate (80%) overnight at 4 °C. The protein precipitates were recovered by centrifugation under similar conditions, and dissolved in same extracting buffer. The crude proteins were dialyzed in deionized water and lyophilized.

### Gel electrophoresis (SDS-PAGE)

The crude protein extract and purified gel filtration fractions were visualized by SDS-PAGE by using 12% Tris/Tricine gels^73^. The electrophoresis was performed at 200 V for 1 hr. The gels were stained with Bio-Safe Coomassie G-250 stain (Bio-Rad) and destained in deionized water.

### Gel filtration chromatography

Gel filtration chromatography was performed by using fast protein liquid chromatography (FPLC) system (NGC Bio-Rad, USA) equipped with multi-wavelength detector and automated fraction collector. A pre-packed gel filtration column Sephacryl S-200 (2.6 × 60 cm, GE Healthcare, USA) was equilibrated with 20 mM Tris/HCl buffer, pH 8.0. The lyophilized crude protein (500 mg) was dissolved in same buffer, filtered through 0.45 µm filter (Millipore, USA), and was applied on to the column. The proteins were eluted at a flow rate of 1 mL/min and absorbance was monitored at 280 nm. The chromatographic fractions of 5.0 mL/tube were collected.

### Reverse phase high performance liquid chromatography

The RP-HPLC was conducted by using Hitachi Model LaChrom equipped with dual pump, UV/VIS multi-wavelength detector, and temperature controlled auto-sampler. A column Aeris Widepore-C4 (250 × 4.6 mm) (Phenomenex, USA) was equilibrated with 0.1% aqueous trifluoroacetic acid (TFA). Proteins were eluted using a gradient of 0.1% TFA-acetonitrile 0–45% in 65 min^74^, and the elution was monitored at 214 nm.

### Modification of cysteine residues

The RP-HPLC purified and lyophilized protein (50 µg) was dissolved in 50 µL of reducing and alkylating (R & A) buffer (Guanidine/HCl 6 M, Tris base 0.2 M, di-sodium EDTA 2 mM) and 5 µL of 2-Mercaptoethanol). The solution was purged with N_2_ gas and incubated at 50 °C for 4 hrs. After cooling the reaction mixture at room temperature, 5 µL of 4-vinylpyridine was added. The sample tube was wrapped with aluminum foil and incubated at 37 °C for 1.5 hr^75^. The reaction was quenched by adding 5 µL of 2-Mercaptoethanol containing 5% acetic acid. The sample was purified by RP-HPLC.

### Trypsin digestion and peptide finger-printing

The pyridylethylated modified protein (50 µg) was dissolved in 100 µL of 50 mM Tris/HCl pH 8.3 buffer. The trypsin enzyme (EC number 3.4.21.), TPCK treated (Tosyl phenylalanyl chloromethyl ketone) was dissolved in the same buffer. Trypsin was used in the ratio of 1:20 w/w (enzyme:substrate) to digest the protein. The digestion was performed for 4 hrs at 37 °C. The reaction was stopped by adding 10 μL of acetic acid^76^. The tryptic digest was separated by RP-HPLC on an Aeris Peptide C18 (3.6 µm, 250 × 4.6 mm) column. The tryptic peptides were eluted using a gradient of 0–40% B (0.1% TFA-acetonitrile) in 50 min. The flow rate was maintained at 1 mL/min and the absorbance was recorded at 214 nm.

### MALDI-TOF mass spectrometry

The mass spectrometric analysis was performed by using Autoflex Speed MALDI-TOF/TOF mass spectrometer (Bruker, USA). The instrument was calibrated by using calibration standard (Bruker, USA). The matrix was prepared by dissolving 10 mg/mL of 3,5-dihydroxybenzoic acid (DHB) in 50% acetonitrile/water. Purified protein was dissolved in 50% acetonitrile, 0.1% TFA. About 2 μL of analyte was mixed with the same volume of matrix solution, applied to MALDI target plate and air dried. The spectra were generated by using Flex-control Software (Bruker, USA).

### *N*-terminal amino acid sequencing

The purified intact protein and tryptic peptides were analyzed for *N*-terminal amino acid sequence in an automated sequencer (PPSQ33A, Shimadzu, USA) equipped with an online PTH phenylthiohydantoin (PTH) analyzer^77^. The samples were mixed in sequencing solvent S4 (37% acetonitrile), and 10 uL sample was loaded onto Polybrene-treated glass fiber disc.

### Homology modelling of nsLTP1

The target sequence consisting of 91 amino acids was submitted to NCBI-Protein BLAST program^54^ using PDB proteins search against BLASTp algorithm for selection of the best template of the closest homologue and to find out the identity, gap region, and similarity between target and template. The best match template sequences as determined by BLASTp were subjected for target-template multiple sequence alignment by Clustal Omega program. Homology modeling was performed by Modeller9 and subsequently the best optimized model was further subjected to energy minimization by AMBER16 software^78^. Finally, several approaches were implemented to evaluate the geometrical and structural consistency of ajwain nsLTP1 homology model. Stereochemical properties of modeled ajwain nsLTP1 protein structure were validated by PROCHECK^68,79^ and the PROSA-web (z-score)^69,80^.

### Molecular docking

Molecular docking was performed between the ajwain nsLTP1 and two lipid molecules; MYR and OLE using the MOE.2016 software package^81^. 3D structures of lipid molecules were constructed with builder module and energy was minimized *via* MMFF94X force field as implemented in the software. Then the model protein structure was also optimized by adding polar hydrogen atoms and partial charges using the MOE.2016 software. After this, energy minimization was performed using default parameters, where the force field was AMBER99. Here, MOE site-finder module was employed to elucidate possible catalytic sites in the target protein from the 3D atomic coordinates of the receptor. It identifies the hydrophobic and hydrophilic regions on the basis of geometric methods. MOE docking software with default parameters was used to obtain the minimum energy conformation of selected ligands in complex with the model protein. Thirty different conformations for each lipid molecule had been generated by MOE. The best conformations of both ligands were used for further molecular interaction analysis.

### Molecular dynamics simulations

To explore the stability and conformational flexibility (global and local) of apo and nsLTP1 complexes, all-atom MD simulations were performed using Amber 16 package^82^. Three systems were prepared and employed for 10 ns time period simulation studies, one for predicting the dynamic stability of modeled ajwain nsLTP1 structure and others for nsLTP1-ligand complexes. The complexes were prepared with Amber ff99SBildn force field for protein and GAFF force field together with AM1-BCC charges for ligands using Antechamber. All the hydrogen atoms were added, and counter-ions (Cl-) were employed to neutralize the highly negative charges of the systems. These systems were finally solvated explicitly in a cubic TIP3P water box and periodic boundary condition with dimensions at least 8 Å away from any protein atoms. The Leap program was used for this purpose.

After the solvation, each system was first structurally relaxed to optimize stereo-chemical restraint and to eliminate atom clashes, minimization was carried out by using pmemd AMBER package. Both steepest descent and conjugate gradient methods were used to minimize potential energy of the systems. Total 1500 steps were carried out. Initially, in 500 steps of minimization harmonic restraint of 25 Kcal/mol Å^2^ were applied on protein and then minimization was followed by gradually relaxing restraint on both protein and counter ions. After minimization systems were heated gradually from 0–300 K up to 40 ps and then subsequently restraint was relaxed and final equilibration was last up to 70 ps.

During equilibration, minimization was switched off and protein backbone atoms were relaxed over multiple steps by gradual decrease in harmonic restraint and at last all restraints were released. The NPT ensemble (isothermal-isobaric) MD productions were performed for 10 ns maintaining temperature at 300 K by Berendsen thermostat and the pressure was kept constant at 1 atm. All bonds involving hydrogen atoms were kept rigid via SHAKE flag (ntc = 2) in order to simplify the calculation and minimize the cost involved in computation power. Particle mesh Ewald method was used to calculate long range electrostatic interactions. For this purpose, the cut-off value for non-bonded interactions was set 9 Å. The analysis and visualization of the trajectories of ajwain nsLTP1 complexes was facilitated using CPPTRAJ included in AMBER 16 and VMD package^83^.

## Supplementary information

Supplementary Info
