# Supplementary material for: Purification and Characterization of a Nonspecific Lipid Transfer Protein 1 (nsLTP1) from Ajwain (Trachyspermum ammi) Seeds
Source: Sci Rep. 2019 Mar 11;9:4148. doi: 10.1038/s41598-019-40574-x (PMC6411740; doi:10.1038/s41598-019-40574-x)

Supplementary Info

Purification and Characterization of a Nonspecific Lipid Transfer Protein 1 (nsLTP1) from Ajwain (*Trachyspermum ammi)* Seeds

Meshal Nazeer[1,2], Humera Waheed[2], Maria Saeed[3], Saman Yousuf Ali[3], M. Iqbal Choudhary[2,3], Zaheer Ul-Haq[3] and Aftab Ahmed[1*]

1. Department of Biomedical and Pharmaceutical Sciences, Chapman University School of Pharmacy, Irvine, CA 92618, USA

2. H.E.J. Research Institute of Chemistry, International Center for Chemical and Biological Sciences, University of Karachi, Karachi 75270, Pakistan

3. Dr. Panjwani Center for Molecular Medicine and Drug Research, International Center for Chemical and Biological Sciences, University of Karachi, Karachi 75270, Pakistan

Table S1: Top-ranked template sequences obtained by BLASTp results.

Rankin g No.	PDB ID	Total Score	Query coverage	Max. Identity (%)	Positives (%)	e-Value
1.	5TVI_V	98.6	100	52	64.84	2.45E-28
2.	2MAL_A	65.9	100	41	52.75	1.90E-15
3.	5LQV_A	64.3	98	40	52.22	7.68E-15
4.	2ALG_A	63.2	98	37	53.85	2.35E-14
5.	1BWO_A	60.1	98	35	54.95	3.82E-13
6.	4XUW_A	58.9	100	38	51.09	1.13E-12
7.	1CZ2_A	58.2	97	34	54.44	1.73E-12
8.	1FK0_A	56.6	100	37	54.84	7.54E-12
9.	1UVA_A	56.6	98	36	53.85	8.02E-12
10.	2N81_A	53.9	100	37	50.54	1.10E-10
11.	1SIY_A	53.1	98	37	50.55	2.05E-10
12.	1MID_A	50.1	98	31	50.55	3.24E-09
13.	1T12_A	47.4	100	36	50	2.78E-08

Figure S1: Multiple Sequence Alignment of the Top-ranked BLASTp Search Sequence templates.

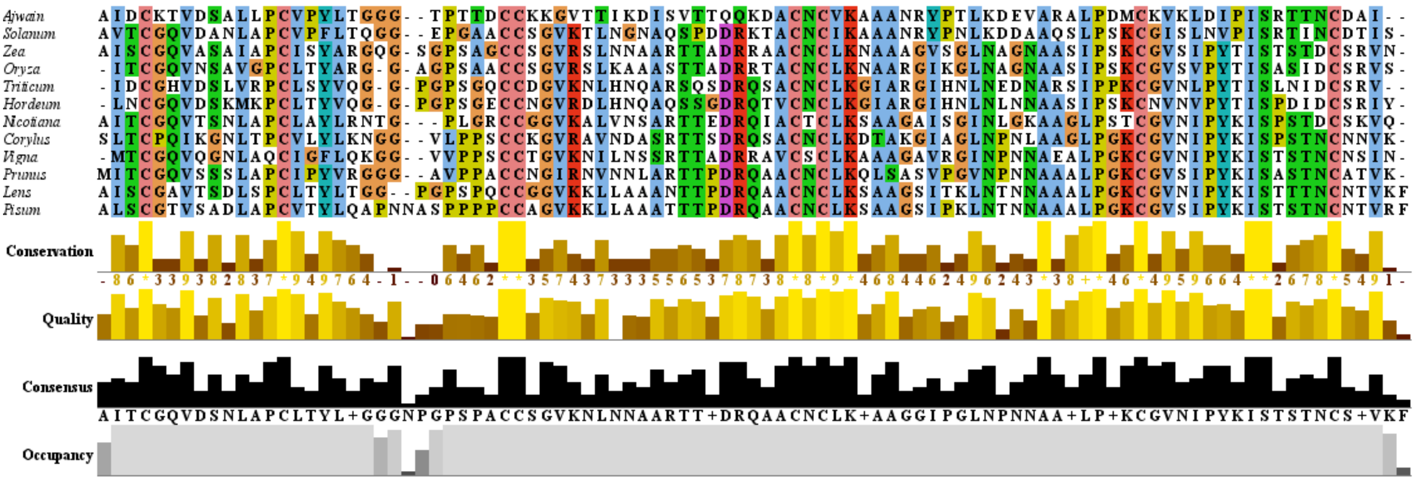

Figure S2: Procheck Result of theoretical model of Ajwain (a) Unrefined theoretical model and (b) Refined theoretical model.

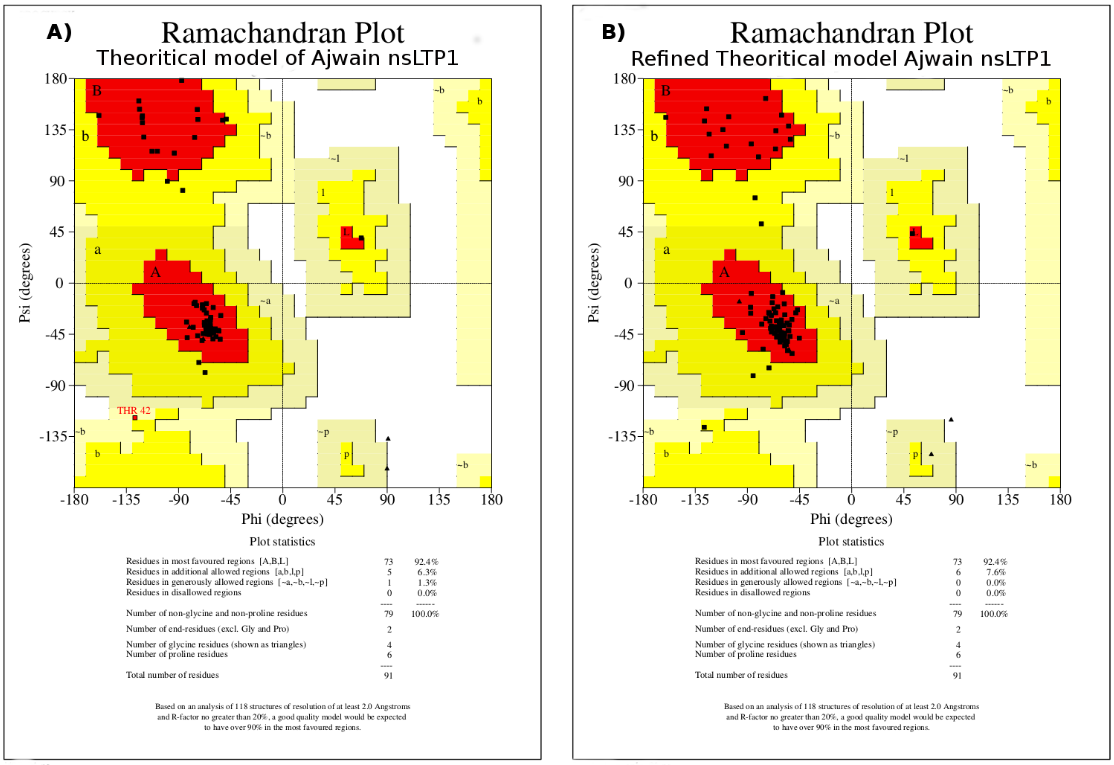

Supplement: Supplementary file 1 — Supplementary Info [file 41598_2019_40574_MOESM1_ESM.pdf]
